# Investigating Bacterial Volatilome for the Classification and Identification of Mycobacterial Species by HS-SPME-GC-MS and Machine Learning

**DOI:** 10.3390/molecules26154600

**Published:** 2021-07-29

**Authors:** Marco Beccaria, Flavio A. Franchina, Mavra Nasir, Theodore Mellors, Jane E. Hill, Giorgia Purcaro

**Affiliations:** 1 Thayer School of Engineering, Dartmouth College, Hanover, NH 03755, USA; tedmellors@gmail.com (T.M.); jane.hill@dartmouth.edu (J.E.H.); gpurcaro@uliege.be (G.P.); 2 Geisel School of Medicine, Dartmouth College, Hanover, NH 03755, USA; mayanasir@gmail.com

**Keywords:** GC-MS, mycobacteria species, machine learning, random forest, SPME, VOCs, features reduction

## Abstract

Species of *Mycobacteriaceae* cause disease in animals and humans, including tuberculosis and leprosy. Individuals infected with organisms in the *Mycobacterium tuberculosis* complex (MTBC) or non-tuberculous mycobacteria (NTM) may present identical symptoms, however the treatment for each can be different. Although the NTM infection is considered less vital due to the chronicity of the disease and the infrequency of occurrence in healthy populations, diagnosis and differentiation among *Mycobacterium* species currently require culture isolation, which can take several weeks. The use of volatile organic compounds (VOCs) is a promising approach for species identification and in recent years has shown promise for use in the rapid analysis of both in vitro cultures as well as ex vivo diagnosis using breath or sputum. The aim of this contribution is to analyze VOCs in the culture headspace of seven different species of mycobacteria and to define the volatilome profiles that are discriminant for each species. For the pre-concentration of VOCs, solid-phase micro-extraction (SPME) was employed and samples were subsequently analyzed using gas chromatography–quadrupole mass spectrometry (GC-qMS). A machine learning approach was applied for the selection of the 13 discriminatory features, which might represent clinically translatable bacterial biomarkers.

## 1. Introduction

The *Mycobacterium* genus consists of over 150 species which can be broadly grouped into fast-growing and slow-growing species or species complexes, based upon physiological, phenotypic and phylogenetic differences [1]. Within *Mycobacteriaceae*, it is possible to distinguish two big families: *Mycobacterium tuberculosis* complex (MTBC), that can cause tuberculosis in several mammals including humans, and the family of non-tuberculous mycobacteria (NTM), which can also infect several mammals, including humans. Both complexes manifest as active disease and dormant disease and while they both mostly present in the lung, infections can also occur elsewhere, such as the skin, spine or eye. Individuals infected with either MTBC or NTMs may generate identical symptoms, however, the antibiotic regimen used to treat is different for TB compared to NTM [2]. Due to the similarity in symptoms and expectations of *M. tuberculosis* being dominant in endemic areas, patients are often mistakenly assumed to have multidrug-resistant tuberculosis when 5% and 30% of suspected cases are caused by NTM. The current, rapid nucleic acid amplification tests, such as GeneXpert™ decrease the diagnostic burden, but this test and others like it do not parse *Mycobacterium* species, which are likely more broadly distributed than currently reported.

Isolation of *Mycobacterium* species from sputum, feces or tissue still represents the gold standard for the diagnosis of the family of *Mycobacteriaceae* [3], but due to the long generation time of some species, a complete diagnosis can take several weeks [4,5]. Moreover, extra-time can be required to identify the specific *Mycobacterium* species for a proper treatment. It is worthy to mention that matrix-assisted laser desorption/ionization (MALDI)-MS technology represents a rapid screening tool, faster than traditional microbiological techniques, capable of distinguishing at species level, even if a previous purification stage is normally required and the instrument represents an important economical investment.

The analysis of volatile organic compounds (VOCs) produced from in vitro cultures and/or ex vivo specimens, represents a viable and cheaper alternative to non-invasive diagnose mycobacteria at the species level [6,7], although large-scale investigations are still needed to validate the identity of the biomarkers [8]. In vitro studies represent an important and easier step to investigate VOCs produced by the specific bacterium under study, thus highlighting possible biomarkers. Nevertheless, these findings need to be then validated in ex vivo and in vivo scenarios. Different analytical techniques are commonly used in VOC analysis, including: sensor-based electronic noses [9,10], microfluidic colorimetric assays [11], selected ion flow tube–mass spectrometry (SIFT-MS) [12], proton transfer reaction mass spectrometry (PTR-MS) [13], ion mobility spectrometry (IMS) with gas chromatographic pre-separation by a multi-capillary column (MCC) [14] and gas chromatography (GC) based techniques coupled to mass spectrometry (MS) [15,16,17]. The ultimate goal would be to transfer the high level of information acquired using the aforementioned instrumentations into an easy and straightforward point-of-care (POC) device to target the biomarkers identified.

In this context, the aim of this work is to investigate the biomarker candidates produced by different mycobacteria species for their classification to contribute to the knowledge needed to develop a reliable POC device, and to provide a reference point for future validation of ex vivo and in vivo studies. Here, a simple, ready-to-use headspace solid-phase microextraction (HS-SPME) GC-MS analytical platform was applied to the putative identification of in vitro biomarkers among seven different mycobacteria species belonging to three different complexes. SPME has been used widely for the analysis of VOCs since its invention in the early 1990s [18,19]. SPME is a simple and effective sample preparation technique which combines sampling, isolation and concentration in a single step. After preconcentration, VOCs from mycobacteria were analyzed by GC-quadrupole (q)MS. The detected analytes were treated with different data processing techniques and the discriminatory capability of the selected volatiles was evaluated. Chemometrics is a well-established aid in the discovery of differences between samples with many variables [20]. In this context, a random forest (RF) machine learning algorithm was applied to select a panel of discriminatory features able to distinguish among different mycobacteria species.

## 2. Materials and Methods

### 2.1. Chemical and Reagents

Hexane was HPLC grade (MilliporeSigma^®^, St. Louis, MO, USA). A mixture of normal alkanes (C_6_–C_20_) was purchased from Supelco (Bellefonte, PA, USA). The mixture of alkanes was injected to calculate the linear retention index (LRI).

### 2.2. Sample Preparation

#### 2.2.1. Bacterial Strains, Culture Conditions

Seven mycobacteria species [*M. abscessus* (Abs)*, M. bollettii* (Bol)*, M. massiliense* (Mas)*, M. avium* (Avi), *M. intracellulare* (Int), *M. chimaera* (Chim) and *M. bovis* (BCG))] were used for all experiments and culture conditions [21]. The considered species belong to three different *Mycobacterium* complexes: (1) *M. tuberculosis* complex (MTB), which includes BCG; (2) *M. avium* complex (MAC), which includes Avi, Int, and Chim; (3) *M. abscessus* complex (MAB), which includes Abs, Mas, and Bol. All species were cultured aerobically (30 mL, 37 °C, 200 rpm shaking) in Difco Middlebrook 7H9 Broth (Becton Dickinson, Franklin Lakes, NJ, USA) containing Tween 80, glycerol and 10% Difco Middlebrook ADC enrichment (BD, Franklin Lakes, NJ, USA) placed into storage at −80 °C until use in this study. Bacterial growth conditions were the same as reported in [21]. Briefly, the bacterial growth was evaluated by measuring the optical density of the culture at 600 nm (OD_600_) (Helios Omega UV/Vis (Thermo Fisher, Waltham, MA, USA). After an OD_600_ of 2.0–2.5 was reached, cultures were transferred to 50 mL conical flasks, placed on ice to stop the metabolism, and centrifuged (8000 rpm, 4 °C, 10 min). In total, 5 mL of culture supernatant was transferred to a 20 mL air-tight glass headspace vial after centrifugation. Six biological replicates were prepared for each sample.

#### 2.2.2. Sample Preparation

The volatile in the headspace of the culture supernatant was extracted using a poly- dimethylsiloxane/carboxen/divinylbenzene (PDMS/Car/DVB) SPME fiber (Supelco, Bellefonte, PA, USA) for 20 min at 37 °C. All samples were agitated at 250 rpm and incubated for 15 min before fiber exposure at the corresponding extraction temperature.

### 2.3. Analytical Instrumentation

All GC-qMS analyses were carried out on a Shimadzu GC2010 and a TQ8050 triple quadrupole mass spectrometer (Shimadzu, Columbia, MD, USA) equipped with an AOC-6000 autosampler. The single quadrupole acquisition mode was exploited on the TQ8050 MS. The SPME fiber was desorbed into the GC inlet at 250 °C for 2 min in splitless mode. Data were acquired by using the GCMS solution software ver. 4.45 (Shimadzu).

The column employed was an SLB-5 ms [(silphenylene polymer, practically equivalent in polarity to poly (5% diphenyl/95% methylsiloxane)], with the following dimensions: 30 m × 0.25 mm ID × 0.25 μm d*_f_* (Supelco, Bellefonte, DE, USA). GC temperature program: 40 °C (hold 1 min) −240 °C at 3 °C/min, then to 350 at 20 °C/min. Helium head pressure (constant linear velocity mode 35 cm/s) was 48 kPa. The MS system was run in full-scan conditions: scan speed 2000 amu/s; mass range 45–400 *m*/*z*. Interface and ion source temperatures: 200 and 250 °C.

### 2.4. Statistical Analysis

Raw GC-MS data sets were post-processed and aligned all together using R package XCMS [22]. A signal-to-noise ratio threshold of 10 was applied for peak detection, extracting the most abundant *m*/*z* for each peak. All statistical analyses were performed using R v3.3.2 (R Foundation for Statistical Computing, Vienna, Austria).

## 3. Results and Discussion

**Figure 1:**
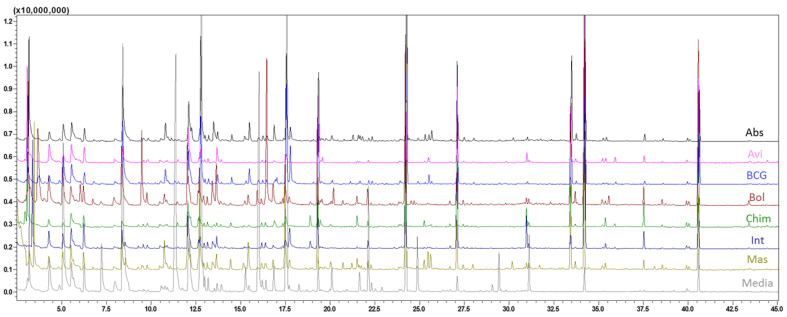
GC-MS total ion current (TIC) chromatogram obtained for the seven different mycobacteria and the medium. Abs—M. *abscessus*; Avi—M. *avium*; BCG—M. *bovis*; Bol—M. *bollettii*; Chim—M. *chimaera*; Int:—M. *intracellulare*; Mas—M. *massiliense*; Media—growth media.

Seven different species of mycobacteria were investigated by HS-SPME-GC-MS, plus the medium for control purpose. Six biological replicates were analyzed for each class. Two replicates (one Avi and one medium) were lost due to a technical problem during sample preparation. Therefore, a total of 46 samples were used in the following analysis. Figure 1 shows a representative VOC total ion chromatogram profile for each species.

**Figure 2:**
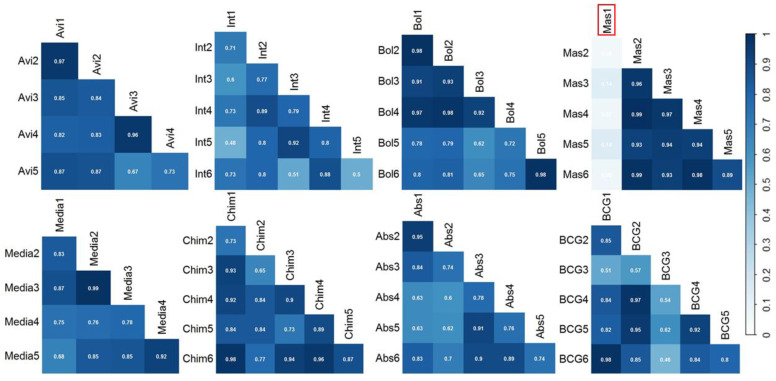
Pearson’s correlation coefficients plot obtained for each mycobacterium specie. Abs—M. *abscessus*; Avi—M. *avium*; BCG—M. *bovis*; Bol—M. *bollettii*; Chim—M. *chimaera*; Int—M. *intracellulare*; Mas—M. *massiliense*; Med—growth media. The number following the abbreviation stands for the replicate number. An outlier—The sample circled in red (Mas1, top right) resulted as an outlier and was excluded from further data elaboration.

The data matrix obtained after alignment, consisting of 879 total features, was first polished by removing common contaminants (e.g., siloxane and phthalates) and then reduced based on a frequency of observation (FOO) cutoff of 50% (i.e., features present in at least three out of six samples within one class were retained for further statistical evaluation), in order to retain the most consistent peaks, resulting in a final data matrix of 667 features. Prior to further statistical analyses, the relative abundance of compounds across chromatograms was normalized using Probabilistic Quotient Normalization (PQN) [23] and log-transformed [15]. On this data matrix, the Pearson’s correlation coefficient was calculated to evaluate the correlation of the overall profile within the biological replicates (Figure 2). Average correlation coefficients within the same class were all above 0.70 (Abs: 0.77; Avi: 0.84; BCG: 0.77; Bol: 0.84; Chim: 0.85; Int: 0.73; Mas: 0.67) except for the Mas species which contained an outlier (Mas1, circled in red in Figure 2) was detected and removed. The removal of the outlier increased the correlation amongst the remaining Mas replicates, with a Pearson coefficient of 0.95. This Pearson test confirmed the high consistency of the sampling and measurements.

**Figure 3:**
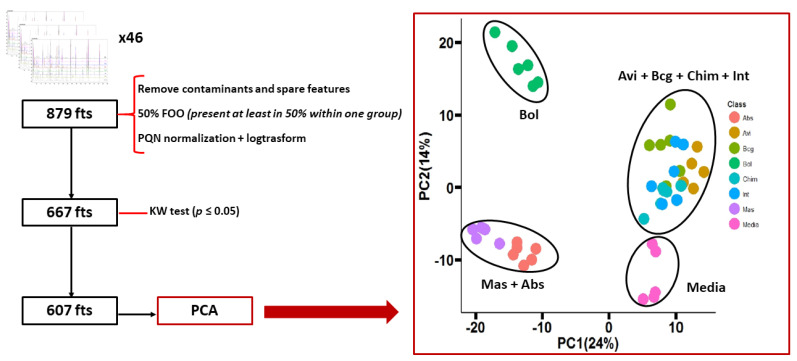
On the left, the data processing flow-chart, from the generation of the data matrix after the alignment to the feature’s reduction after KW test (*p* ≤ 0.05). On the right, the PCA of seven mycobacteria species + medium plotting the 607 volatile significant features.

To test for statistical significance, the Kruskal–Wallis (KW) test [24], with post-hoc Dunn test and Benjamini–Hochberg (BH) correction [25] to minimize the false discovery rate, was used. All features not significantly different (*p* > 0.05) between the different mycobacteria species and the medium were removed, obtaining a panel of 607 features. This panel was used for a first evaluation of the discriminatory capability of the VOC profile. The principal component analysis (PCA) obtained, along with the flowchart to reduce to the panel of 607 features, are reported in Figure 3**.** The PCA showed a rather low total variance of 38%. The discrimination between the groups was not very clear. Only the medium and the group of Bol were well separated from the others in the PCA space, while the other species were spread on the PCA space giving two different clusters: one containing Mas + Abs (bottom left of the PCA in Figure 3) and another big cluster containing the other four mycobacteria species, Avi + BCG + Chim + Int (the top-right of the PCA in Figure 3).

Due to the high dimensional nature of -omics data, it is essential that machine algorithms are selected which can handle cases when the number of features far outweigh the number of samples [26]. Moreover, these algorithms need to also be able to handle highly correlated features (multicollinearity). In this context, to improve the discrimination capability of the overall classification, the random forest (RF) algorithm was applied to select and retain the most discriminatory features. RF is a machine learning algorithm that works by generating many classification trees, using randomly selected subsamples of both features and data points. Features are ultimately selected based on which variables best divides the data according to class at each split [27]. A six-classes analysis was carried out. Features were ranked according to their mean decrease accuracy. In total, 13 features were selected to maximize the accuracy of the model (Table 1).

**Figure 4:**
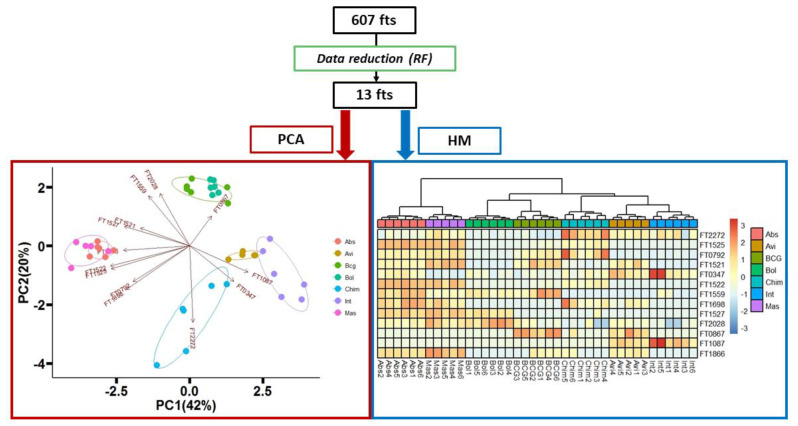
On the top, data feature reduction using RF algorithm, leading to generation of a panel of 13 most discriminatory features. On the bottom left, PCA of seven mycobacteria species plotting the panel of 13 features. On the bottom right, the dendrogram (top of the heatmap) depicts the relatedness amongst samples. Color scheme for samples is based on the mycobacteria species. Legenda: FT0347, 2-Butanol, 2,3-dimethyl-; FT1087, Hexanal; FT0867, Furan, 2-butyl-; FT1559, Furan, 2-methyl-3-(methylthio)-; FT0792, Phenylacetaldehyde; FT1522, unknown; FT1525, (*Z*)-2-Hexenal diethyl acetal; FT1527, Decanal; FT1698, 2-Nonenoic acid, methyl ester; FT1521, unknown; FT1866, unknown; FT2028, unknown; FT2272, Ethyl 4-*t*-butylbenzoate. Mycobacteria species and complex are reported in Section 2.2.1.

The samples were visualized again using a PCA and heatmap (HM) but limiting the data matrix to the selected 13 features (Figure 4). The variance explained by the 2D-PCA, considering both the first and the second principal components, was improved from 38% to 62%. All the different mycobacteria species were well discriminated, resolving most of the misclassifications present in the PCA built with the panel of 607 features (example the misclassification of sample Int5, Figure 3). Moreover, proximity-based on the complex they belong to, namely MTB, MAB and MAC were also depicted both on the PCA and heatmap. On the right side of the heatmap and on the bottom of the PCA, we can observe the proximity of Int, Avi and Chim, belonging to the MAC complex. On the left side, Abs and Mas are clustering together, as both belong to the MAB complex. While Bol, still part of the MAB complex, is clustered in a separate branch in proximity to BCG, which is the only member of the MTB complex. The taxonomic of the MAB complex has been the subject of intense investigation since a clear classification is still not reach, in fact, Leao et al. suggested to combine the species MAS and ABS [28]. However, it has been clearly demonstrated that the two groups are not completely homogeneous, especially in terms of susceptibility to macrolides [29]. Our results confirmed this difficulty, although discrimination can be observed between Mas and Abs. Moreover, these two species can be clearly differentiating from BOL (Figure 4). However, it is not clear why Bol was in such proximity with BCG, although perfectly separated, at a higher distance compared to the other species belonging to the same complex.

**Figure 5:**
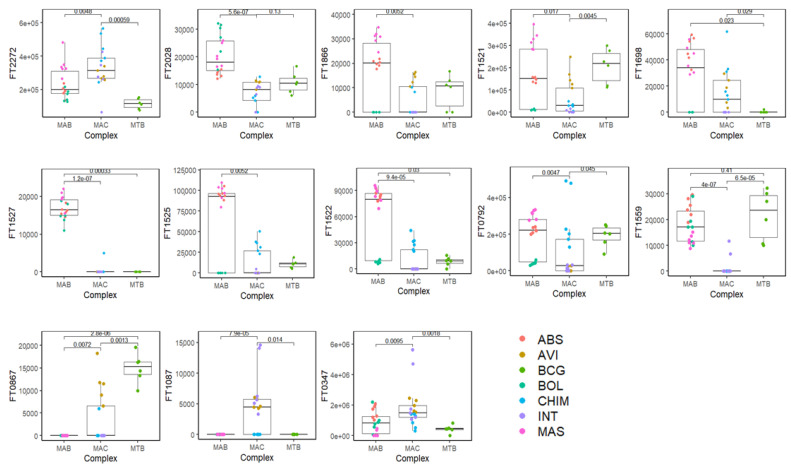
Bar-plot of 13 discriminatory volatile features (FT). Legenda: FT0347, 2-Butanol, 2,3-dimethyl-; FT1087, Hexanal; FT0867, Furan, 2-butyl-; FT1559, Furan, 2-methyl-3-(methylthio)-; FT0792, Phenylacetaldehyde; FT1522, unknown; FT1525, (*Z*)-2-Hexenal diethyl acetal; FT1527, Decanal; FT1698, 2-Nonenoic acid, methyl ester; FT1521, unknown; FT1866, unknown; FT2028, unknown; FT2272, Ethyl 4-*t*-butylbenzoate. Mycobacteria species and complex are reported in Section 2.2.1.

The panel of the most discriminatory features (*n* = 13) is reported in Table 1. The table contains the original number of features (FT), the name of the VOC, experimental LRI and the LRI reported in the literature along with the MS similarity match with the library. The compounds were putatively identified based on the combination of a dual filter: the MS similarity with the NIST17 library (≥80%) and the experimental linear retention index (LRI) within a ±5 range compared to the literature on the same or equivalent column phase. Compounds that did not match with the previous filters were assigned as unknown. Considering the combination of the filters used for the identification of the discriminatory features (MS similarity + LRI), it was possible to name 8 out of 13 volatile molecules (Table 1). Figure 5 shows the box-plot of each discriminatory features (FT) and how they were discriminatory among and within the three mycobacteria complex. FT1559 (Furan, 2-methyl-3-(methylthio)-) and FT0867 (Furan, 2-butyl-) were discriminant among each *Mycobacterium* complex (MAB, MAC, and MTB), while FT0792 (Phenylacetaldehyde), FT1866 (unknown) and FT1521 (unknown) were discriminant within either the MAB complex (resolving the overlapping between MAS and ABS), or the MAC complex, well separating the classes of the mycobacteria belonging to these complexes individually.

## 4. Conclusions

In the present study, clinical isolates of seven mycobacteria species were analyzed using HS-SPME/GC-qMS and a panel of molecules was selected for species-level discrimination. Although more sophisticated analytical tools in combination with SPME are often used in VOCs analysis, e.g., multidimensional comprehensive GC and/or high-resolution MS, GC-qMS proved to still be an effective simple(r) tool to discriminate among different bacteria strains based on their volatile profiles. The fusion of GC-qMS with advanced machine learning algorithms (i.e., random forest) for model building and feature selection results a powerful marriage to unveil the hidden structure of complex metabolite profiles. A panel of 13 features was obtained after the RF model and this panel could be used to distinguish among mycobacteria classes belonging to different complexes. In total, 8 out of 13 discriminatory volatile molecules were also tentatively identified based on MS similarity and LRI. These results provide a proof-of-concept that mycobacteria’s VOCs profiles hold a diagnostic utility for clinical applications in differentiating mycobacteria at the species level, even though more research testing in vivo cases should be performed to confirm their translatability.

## Figures and Tables

**Table 1:** Panel of 13 discriminatory features of seven mycobacteria species after random forest data reduction. Number of features, chemical name, CAS number, MS similarity (%), LRI calculated, LRI in NIST library (LRI lib) and retention time (Rt) are also reported.

FT n.	VOC	CAS	MS%	LRI	LRI lib	Rt
FT0347	2-Butanol, 2,3-dimethyl-	594-60-5	83	649	645	2.31
FT1087	Hexanal	66-25-1	94	800	801	5.31
FT0867	Furan, 2-butyl-	4466-24-4	83	888	890	7.98
FT1559	Furan, 2-methyl-3-(methylthio)-	63012-97-5	84	942	946	10.23
FT0792	Phenylacetaldehyde	122-78-1	85	1037	1045	14.50
FT1522	unknown			1074		16.27
FT1525	(*Z*)-2-Hexenal diethyl acetal	87383-46-8	81	1078	1077	16.49
FT1527	Decanal	112-31-2	81	1171	1187	20.86
FT1698	2-Nonenoic acid, methyl ester	111-79-5	81	1189	1191	21.79
FT1521	unknown			1270		25.51
FT1866	unknown			1326		28.03
FT2028	unknown			1462		33.85
FT2272	Ethyl 4-*t*-butylbenzoate	5406-57-5	80	1498	1487	35.42

## Data Availability

The data presented in this study are available on request from the corresponding authors.

## Sample Availability

Samples are not available from the authors.
